# Side-Chain Immune Oxysterols Induce Neuroinflammation by Activating Microglia

**DOI:** 10.3390/ijms242015288

**Published:** 2023-10-18

**Authors:** Yonghae Son, In-Jun Yeo, Jin-Tae Hong, Seong-Kug Eo, Dongjun Lee, Koanhoi Kim

**Affiliations:** 1 Department of Pharmacology, School of Medicine, Pusan National University, Yangsan 50612, Gyeongnam, Republic of Korea; squall0211@hanmail.net; 2 College of Pharmacy, Kyungpook National University, 80 Daehak-ro, Buk-gu, Daegu 41566, Gyeongbuk, Republic of Korea; section18@naver.com; 3 College of Pharmacy and Medical Research Center, Chungbuk National University, Osong-eup, Heungdeok-gu, Cheongju 28160, Chungbuk, Republic of Korea; jinthong@chungbuk.ac.kr; 4 College of Veterinary Medicine and Bio-Safety Research Institute, Jeonbuk National University, Iksan 54596, Jeonbuk, Republic of Korea; vetvirus@chonbuk.ac.kr; 5 Department of Convergence Medicine, School of Medicine, Pusan National University, Yangsan 50612, Gyeongnam, Republic of Korea

**Keywords:** immune oxysterol, interleukin-1β, microglia, neuroinflammation

## Abstract

In individuals with Alzheimer’s disease, the brain exhibits elevated levels of IL-1β and oxygenated cholesterol molecules (oxysterols). This study aimed to investigate the effects of side-chain oxysterols on IL-1β expression using HMC3 microglial cells and ApoE-deficient mice. Treatment of HMC3 cells with 25-hydroxycholesterol (25OHChol) and 27-hydroxycholesterol (27OHChol) led to increased IL-1β expression at the transcript and protein levels. Additionally, these oxysterols upregulated the surface expression of MHC II, a marker of activated microglia. Immunohistochemistry performed on the mice showed increased microglial expression of IL-1β and MHC II when fed a high-cholesterol diet. However, cholesterol and 24s-hydroxycholesterol did not increase IL-1β transcript levels or MHC II expression. The extent of IL-1β increase induced by 25OHChol and 27OHChol was comparable to that caused by oligomeric β-amyloid, and the IL-1β expression induced by the oxysterols was not impaired by polymyxin B, which inhibited lipopolysaccharide-induced IL-1β expression. Both oxysterols enhanced the phosphorylation of Akt, ERK, and Src, and inhibition of these kinase pathways with pharmacological inhibitors suppressed the expression of IL-1β and MHC II. The pharmacological agents chlorpromazine and cyclosporin A also impaired the oxysterol-induced expression of IL-1β and upregulation of MHC II. Overall, these findings suggest that dysregulated cholesterol metabolism leading to elevated levels of side-chain oxysterols, such as 25OHChol and 27OHChol, can activate microglia to secrete IL-1β through a mechanism amenable to pharmacologic intervention. The activation of microglia and subsequent neuroinflammation elicited by the immune oxysterols can contribute to the development of neurodegenerative diseases.

## 1. Introduction

Recent studies shed light on the intricate relationship between high cholesterol levels and Alzheimer’s disease (AD) [1,2,3,4]. Cholesterol, being highly susceptible to oxidation, undergoes enzymatic oxidation or autoxidation when exposed to oxidizing agents, resulting in the formation of oxysterols [5]. The brain, being the most cholesterol-rich organ, exhibits distinct oxysterol profiles in AD patients compared to healthy brains [2]. In a healthy brain, the predominant oxysterol is 24s-hydroxycholesterol (24sOHChol), followed by 25-hydroxycholesterol (25OHChol) and 27-hydroxycholesterol (27OHChol). Conversely, in AD patients’ brains, certain oxysterols, including 25OHChol, 27OHChol, 7-ketocholesterol (7K), 7α-hydroxycholesterol (7αOHChol), and 7β-hydroxycholesterol (7βOHChol), show elevated levels, while 24sOHChol levels decrease [6,7]. Furthermore, inflammatory mediators such as interleukin-1 β (IL-1β), monocyte chemotactic protein-1, and matrix metalloprotease-9 exhibit significant upregulation in the brains of AD patients [6,7]. These compelling findings suggest a potential connection between cholesterol metabolism, oxysterol production, and inflammation in the pathogenesis of AD.

Microglia, the resident immune cells in the brain, play a pivotal role in the neuroinflammatory response. These cells exhibit a distinctive morphology characterized by a cell body (soma) adorned with elongated, ramified processes [8,9]. However, in reactive conditions such as insults, injuries, or diseases, microglial processes may undergo significant alterations in number, length, and branching complexity, accompanied by variations in cell body size and shape [10]. Beyond morphological changes, activated microglia secrete inflammatory cytokines at the site of inflammation, including IL-1β, and display elevated levels of MHC class II (MHC II) molecules [11]. IL-1β is implicated in neuroinflammation and various cellular events, encompassing proliferation, differentiation, and apoptosis, thereby exerting pleiotropic effects on immune cells within the brain [12]. Microglial activation and neuroinflammation represent prominent features of AD, with emerging evidence suggesting a dual role for microglia in the disease’s pathogenesis [13]. On one hand, microglia form barriers aimed at eliminating pathogens and damaged cells, while paradoxically, they may contribute to neuroinflammation and neuronal damage, contingent upon the context and specific mechanisms involved [14,15]. The activation of microglia, coupled with the release of inflammatory mediators and alterations in oxysterol profiles, could potentially amplify neuroinflammation in AD.

25OHChol and 27OHChol can be categorized as immune oxysterols, also known as immunosterols, due to their involvement in inflammatory immune responses, as they activate immune cells and enhance the production of inflammatory mediators [16,17,18]. However, it is yet to be determined whether these oxysterols induce neuroinflammatory responses. In this study, we investigated the effects of side-chain oxysterols on the expression of IL-1β and microglial activation. We utilized HMC3 microglial cells and apolipoprotein E (ApoE)-deficient mice to explore whether oxysterol molecules formed in a cholesterol-rich environment play a fundamental role in neuroinflammation.

## 2. Results

### 2.1. Increased Expression of IL-1β in Microglia following Treatment with 25OHChol, 27OHChol, Aβ, and LPS

**Figure 1:**
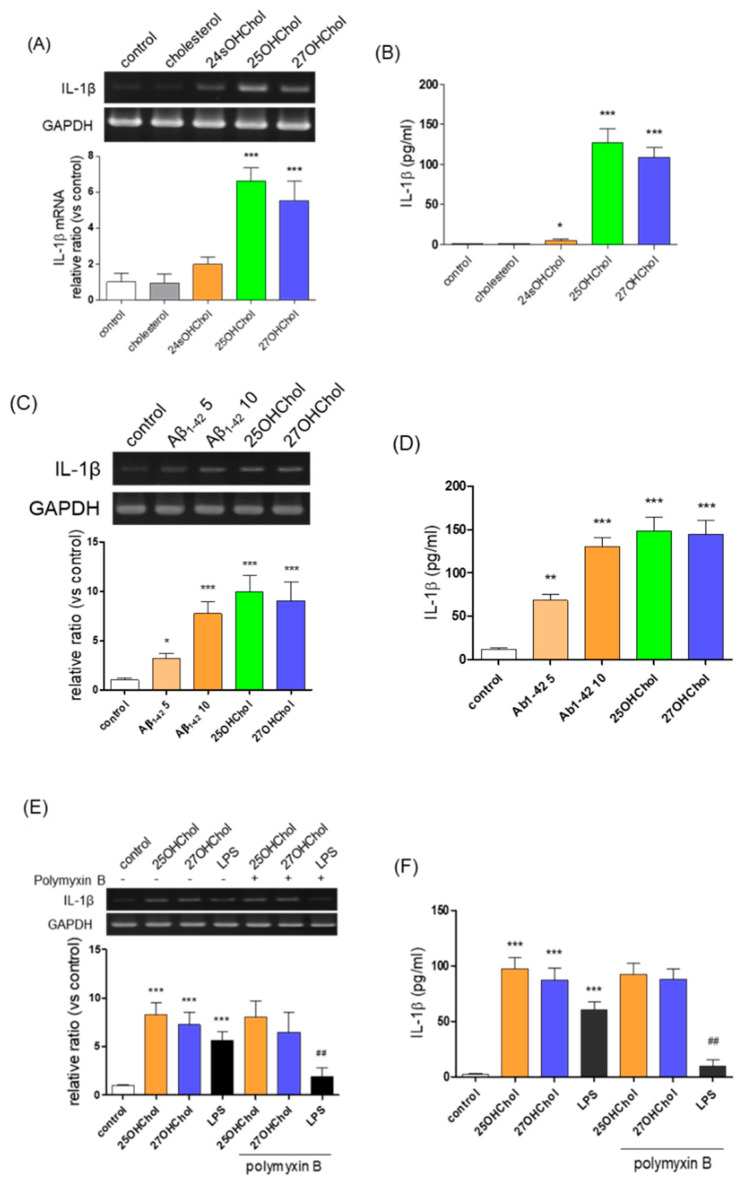
The effects of side chain oxysterols, Aβ_1–42,_ and LPS on the microglial expression of IL-1β. HMC3 cells (1 × 10^6^ cells/dish) were treated for 48 h with cholesterol or the indicated oxysterols (1 μg/mL each). (**A**) IL-1β transcripts were detected by RT-PCR and levels of IL-1β transcripts were analyzed by qPCR. The y-axis values represent fold increases in IL-1β mRNA levels normalized to GAPDH levels relative to that of the untreated microglia (control). * *p* < 0.05 vs. control; *** *p* < 0.001 vs. control. (**B**) The concentrations of IL-1β secreted from cells to the culture medium were quantitatively detected by ELISA. *** *p* < 0.001 vs. control. (**C**) The cells were stimulated with 5 and 10 μM of Aβ_1–42_ or with 25OHChol or 27OHChol (1 μg/mL each) for 48 h. Using total RNA isolated from cells, IL-1β transcripts were detected by RT-PCR, and levels of IL-1β transcripts were assessed by qPCR. (**D**) The amounts of IL-1β released into culture media were quantitatively measured by ELISA. *** *p* < 0.001 vs. control; ** *p* < 0.01 vs. control. Data are expressed as the mean ± SD (*n* = 3 replicates for each group). (**E**) The cells were treated with the indicated oxysterols (1 μg/mL each) and LPS (100 ng/mL) in the absence or presence of polymyxin B (10 μg/mL) for 48 h. IL-1β transcripts were detected and assessed by RT-PCR and qPCR, respectively, and (**F**) the amounts of IL-1β secreted to culture media were measured by ELISA. *** *p* < 0.001 vs. control; ^##^ *p* < 0.01 vs. LPS. Data are representative of three independent experiments and are expressed as mean ± SD (*n* = 3 replicates for each group).

To determine which molecules are involved in microglial activation under hypercholesterolemic conditions, we investigated the effects of cholesterol, 24sOHChol, 25OHChol, and 27OHChol on IL-1β expression using the human microglial clone 3 (HMC3) cell line. Treatment with 25OHChol and 27OHChol significantly increased IL-1β transcript levels (Figure 1A) and promoted IL-1β secretion from microglia (Figure 1B). In contrast, cholesterol or 24sOHChol had no discernible effect. We also assessed the impact of aggregated Aβ on IL-1β expression in HMC3 cells. After a 48 h treatment with 5 μM human β-amyloid peptide (1–42) (Aβ_1–42_), we observed elevated levels of IL-1β transcript and secreted IL-1β protein. These increases were further potentiated in the presence of 10 μM Aβ_1–42_, reaching levels comparable to those induced by 1 μg/mL of 25OHChol and 27OHChol (Figure 1C,D). However, this heightened IL-1β expression was accompanied by a decrease in cell viability (Supplementary Figure S1). To explore potential synergism between Aβ and oxysterols, we conducted an experiment wherein cells were treated with sub-cytotoxic concentrations of Aβ in the presence of 25OHChol and 27OHChol (Supplementary Figure S2). Both oxysterols increased IL-1β transcript levels and enhanced IL-1β secretion. Notably, the expression of IL-1β induced by 25OHChol and 27OHChol remained unaffected by treatment with 5 μM Aβ_1–42_, suggesting that side-chain oxysterols and Aβ independently induce IL-1β expression.

We also explored the impact of polymyxin B, a commonly employed agent to mitigate the effects of endotoxins, on IL-1β expression (Figure 1E,F). The induction of IL-1β expression in HMC3 microglial cells by lipopolysaccharide (LPS; derived from *E. coli* K12) was markedly diminished in the presence of polymyxin B, indicating its inhibitory effect on LPS-induced IL-1β expression. However, treatment with polymyxin B had no discernible effect on IL-1β expression induced by 25OHChol and 27OHChol. These findings exclude the potential contribution of contaminating endotoxins, if any, in the IL-1β expression triggered by oxysterols. In summary, the aforementioned results suggest that the mechanism underlying the induction of IL-1β expression by 25OHChol and 27OHChol differs from that induced by Aβ or LPS, thereby indicating distinct pathways of inflammation.

### 2.2. Concentration and Time-Course Effects of 25OHChol and 27OHChol on IL-1β Expression

**Figure 2:**
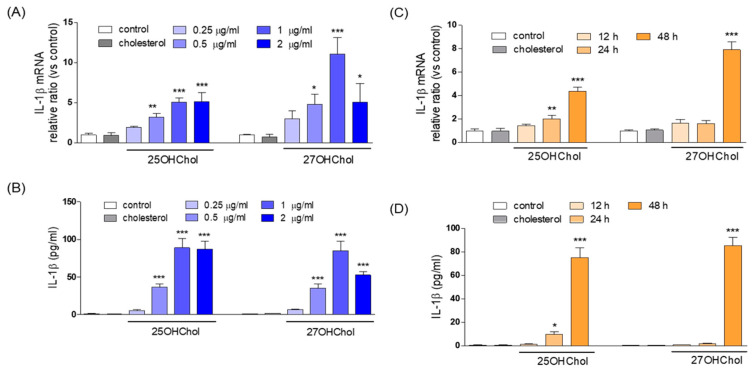
Concentration and time-course effects of 25OHChol and 27OHChol on IL-1β expression. Microglia were treated with the indicated concentrations of 25OHChol and 27OHChol for 48 h. Levels of IL-1β transcripts were assessed by qPCR (**A**), and concentrations of IL-1β protein secreted to the medium were quantitatively detected by ELISA (**B**). *** *p* < 0.001 vs. control; ** *p* < 0.01 vs. control; and * *p* < 0.05 vs. control. Data are expressed as the mean ± SD (*n* = 3 replicates for each group). Following treatment of microglia with 25OHChol and 27OHChol (1 μg/mL each) for the indicated periods, levels of IL-1β transcripts and secreted IL-1β protein were measured by qPCR (**C**) and by ELISA (**D**), respectively. *** *p* < 0.001 vs. control; ** *p* < 0.01 vs. control; and * *p* < 0.05 vs. control. Data are expressed as the mean ± SD (*n* = 3 replicates for each group).

We investigated the concentration- and time-course effects of 25OHChol and 27OHChol on IL-1β expression (Figure 2). Concentration effects were evaluated following treatment with sub-cytotoxic concentrations of each oxysterol for 48 h. Viability assessments demonstrated that 25OHChol and 27OHChol did not adversely affect HMC3 cell viability at concentrations up to 2 μg/mL (Supplementary Figure S1). Notably, 25OHChol increased IL-1β transcript levels at 0.5 μg/mL, with a plateau observed at 1 μg/mL, and sustained at 2 μg/mL of 25OHChol. Similarly, 27OHChol elevated IL-1β levels at 0.5 μg/mL, peaking at 1 μg/mL and decreasing thereafter (Figure 2A). Both oxysterols enhanced IL-1β secretion from microglia in a manner consistent with their transcriptional regulation (Figure 2B).

Time-course effects were investigated following treatment with 1 μg/mL of each oxysterol, representing the concentration eliciting maximal IL-1β expression. Both 25OHChol and 27OHChol exhibited nearly identical time-course effects on IL-1β expression at both the transcript and protein levels. Specifically, 27OHChol increased IL-1β gene transcripts and IL-1β protein secretion 48 h after treatment. In contrast, treatment with 25OHChol initiated an increase in both IL-1β gene transcripts and IL-1β protein secretion after 24 h, with further enhancement at 48 h (Figure 2C,D). These findings suggest that 25OHChol and 27OHChol induce IL-1β expression in a similar but distinct temporal pattern.

### 2.3. Involvement of Multiple Signaling Pathways in the Expression of IL-1β Induced by 25OHChol and 27OHChol

**Figure 3:**
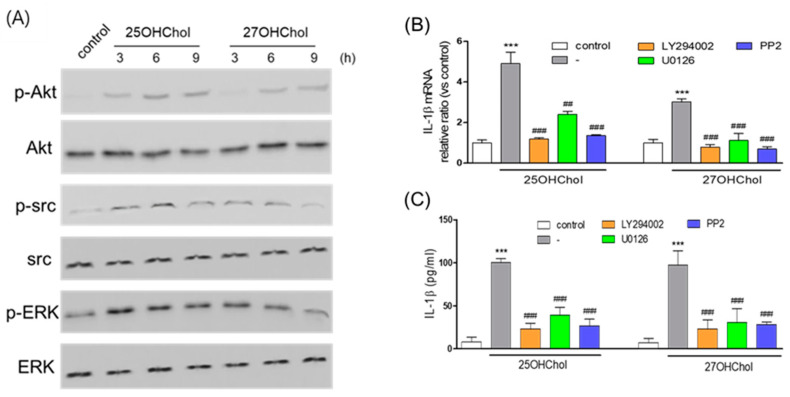
Effects of protein kinase inhibitors on IL-1β expression induced by the oxysterols. (**A**) Cell lysates were obtained after treatment of microglia with 25OHChol or 27OHChol (1 μg/mL) for the indicated periods. Following the determination of protein concentrations, an equal amount of protein was analyzed by Western blotting using Abs against phosphorylated and unphosphorylated forms of Akt, ERk1/2, and Src. Results are representative of three independent experiments. Microglia were treated with 25OHChol and 27OHChol for 48 h in the absence or presence of the indicated kinase inhibitors (10 μM each). Levels of IL-1β transcripts were assessed by qPCR (**B**), and the amounts of IL-1β protein released from cells were measured by ELISA (**C**). *** *p* < 0.001 vs. control; ^###^ *p* < 0.001 vs. 25OHChol or 27OHChol; and ^##^ *p* < 0.01 vs. 25OHChol. Data are expressed as the mean ± SD (*n* = 3 replicates for each group).

We investigated the activation of Akt, ERK1/2, and Src by assessing their phosphorylation in response to oxysterols through immunoblotting. Phosphorylation of Akt, ERK1/2, and Src was significantly augmented by 25OHChol and 27OHChol treatment (Figure 3A). Specifically, Akt phosphorylation exhibited increases at 3 h and 6 h following treatment with 25OHChol and 27OHChol, respectively, with this effect persisting for up to 9 h. Furthermore, treatment with 25OHChol and 27OHChol also induced notable phosphorylation of Src and ERK. To probe the involvement of protein kinases in IL-1β expression, we employed pharmacological inhibitors. Treatment with LY294002 (a PI3K inhibitor), U0126 (an ERK1/2 activation inhibitor), and PP2 (an Src kinase inhibitor) resulted in downregulated IL-1β transcription induced by 25OHChol and 27OHChol (Figure 3B). These inhibitors also significantly suppressed IL-1β secretion from microglia (Figure 3C). Notably, the inhibitory effects of the kinase inhibitors did not exert cytotoxicity, as evidenced by unchanged cell viability (Supplementary Figure S1). Collectively, our findings suggest that pathways involving these kinases contribute to the effects induced by 25OHChol and 27OHChol.

### 2.4. Upregulated Expression of MHC II on the Cell Surface following Stimulation with 25OHChol and 27OHChol

**Figure 4:**
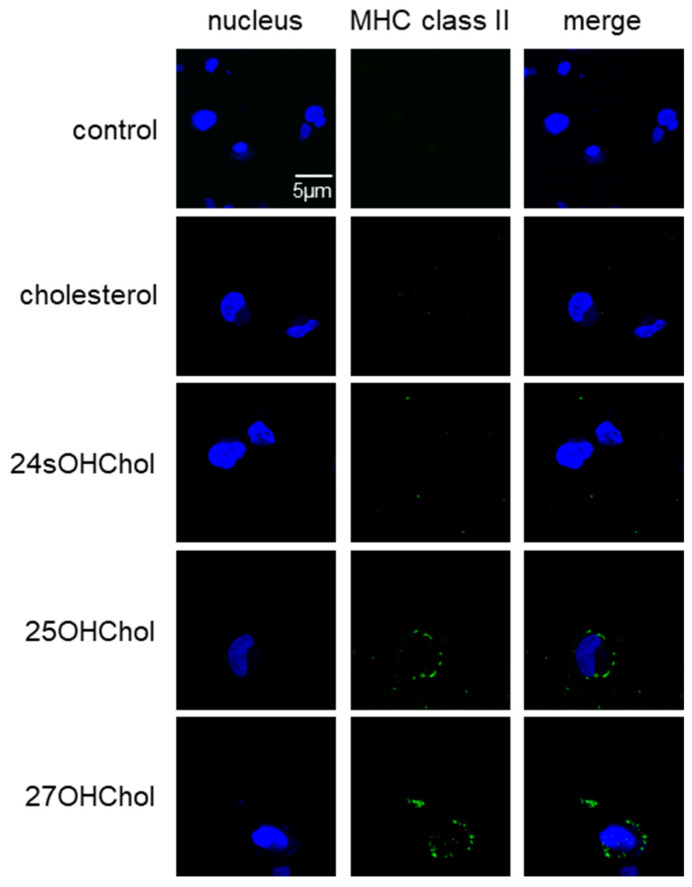
Enhanced MHC II expression on the microglial surface following treatment with side-chain oxysterols. HMC3 microglial cells were seeded on coverslips and treated for 48 h with cholesterol, 24sOHChol, 25OHCho1, and 27OHChol (1 μg/mL each). The cells were immunostained with a fluorescence-conjugated antibody against MHC II (green). The nuclei were stained with DAPI (blue). The fluorescence signal representing MHC II was visualized using a confocal microscope at a magnification of 200×. The data presented are representative of three independent experiments.

We assessed the effects of oxysterols on the expression of MHC II molecules, which serve as an activation marker for microglia, in the HMC3 cell line (Figure 4). Our observations revealed a notable increase in MHC II immunoreactivity (green fluorescence) in microglia exposed to 25OHChol and 27OHChol. Conversely, we could not detect any significant MHC II response following treatment with cholesterol or 24sOHChol. These findings strongly suggest the activation of microglia in the presence of 25OHChol and 27OHChol. Furthermore, we investigated the impact of protein kinase inhibition on MHC II expression (Supplementary Figure S3). The MHC II immunoreactivity, which exhibited an increase after treatment with 25OHChol and 27OHChol, was attenuated in the presence of LY294002, U0126, or PP2. These results emphasize the involvement of PI3K, ERK, and Src signaling pathways in mediating microglial activation induced by 25OHChol and 27OHChol.

### 2.5. Enhanced Microglial Expression of IL-1β and MHC II in ApoE-Deficient Mice

**Figure 5:**
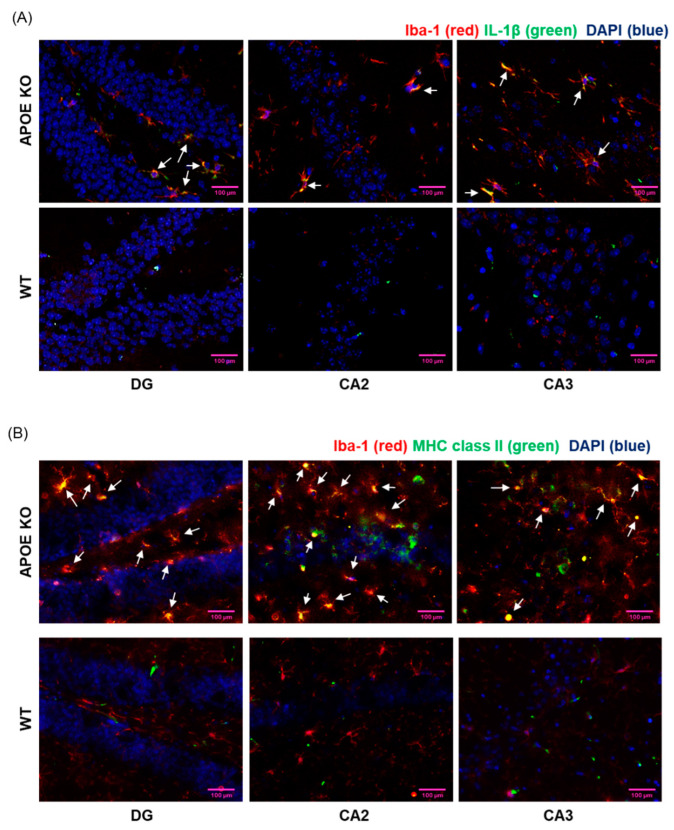
Microglial activation in the brain of ApoE-deficient mice fed HFD (*n* = 10). The brain sections prepared from wild-type (WT) and ApoE-deficient (ApoE-KO) mice were immunostained with fluorescence-conjugated antibodies. After staining the nuclei with DAPI (blue), the fluorescence was visualized by confocal microscopy. (**A**) The sections were labeled with antibodies against anti-Iba-1 (red) and anti-IL-1β (green). Co-localized regions are indicated by white arrows. (**B**) The sections were labeled with anti-Iba-1 (red) and anti-MHC II (green) antibodies. Co-localized images are indicated by white arrows. The images presented are representative of three samples, and the results are representative of three independent experiments. Scale bars represent 100 μm.

To investigate the correlation between elevated cholesterol levels and microglial activation, we assessed the expression of IL-1β and MHC II in ApoE-deficient mice subjected to a high-cholesterol diet. Immunostaining was conducted on murine brain tissue utilizing fluorescence-conjugated antibodies targeting Iba-1 (a microglial marker; red signal) and IL-1β (green signal) (Figure 5A). We observed substantial immunoreactivity of the proinflammatory cytokine IL-1β in the hippocampus of mice exposed to a high-cholesterol diet, while such immunoreactivity was absent in the hippocampus of wild-type mice. Notably, the IL-1β expression coincided with microglial immunoreactivity (indicated by white arrows) in the dentate gyrus (DG), cornu ammonis 2 (CA2), and cornu ammonis 3 (CA3) regions of the hippocampus in ApoE-deficient mice. Additionally, IL-1β released signals were detected in hippocampal tissue (Supplementary Figure S4). Furthermore, we observed MHC II-positive activated microglia (white arrows) in the brains of ApoE-deficient mice (Figure 5B). These findings suggest that the consumption of a high-cholesterol diet induces microglial activation and IL-1β expression in the hippocampus of ApoE-deficient mice.

### 2.6. Suppressive Effects of Cyclosporin A (CsA) and Chlorpromazine (CPZ) on the Expression of IL-1β and the Activation of Microglia Induced by 25OHChol and 27OHChol

**Figure 6:**
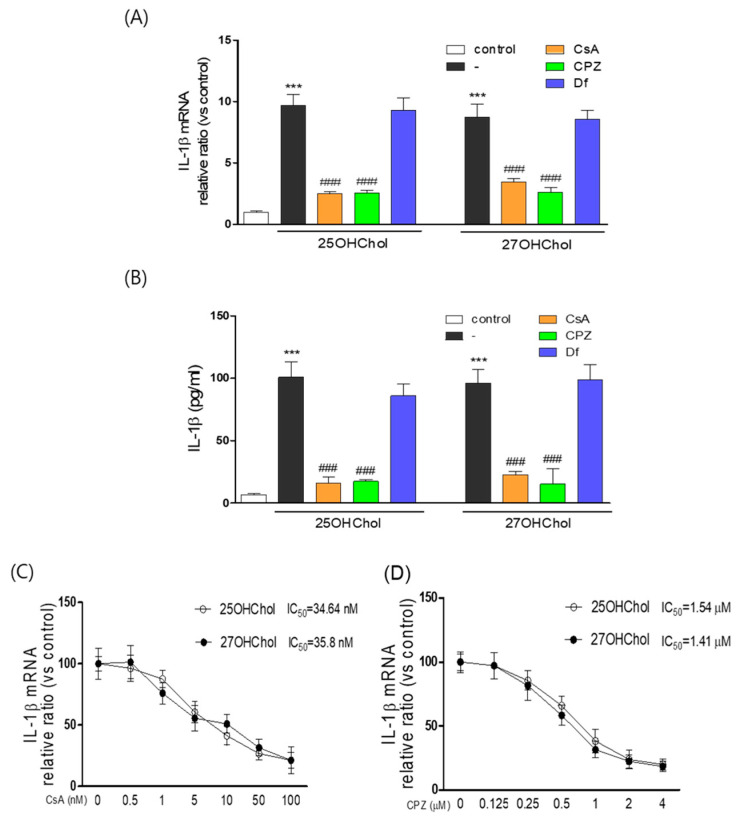
Impaired microglial expression of IL-1β by treatment with CsA and CPZ. Microglial cells (1 × 10^6^ cell/dish) were stimulated for 48 h with 1 μg/mL of the indicated oxysterol in the presence of CPZ (2 μM), CsA (50 nM), or Df (25 μg/mL). (**A**) Total RNA was isolated from the cells, and levels of IL-1β transcripts were measured by qPCR. (**B**) The amounts of secreted IL-1β in culture media were quantitatively detected by ELISA. *** *p* < 0.01 vs. control; ^###^ *p* < 0.01 vs. oxysterols. After treatment of the cells for 48 h with 1 μg/mL of 25OHChol or 27OHChol in the presence of the indicated concentrations of CsA (**C**) and CPZ (**D**), IL-1β transcript levels were assessed by qPCR to calculate the IC_50_ values. The results are representative of three independent experiments.

We previously reported that CsA and diclofenac (Df) inhibit inflammatory responses induced by oxysterols in monocytic cells [19,20]. Therefore, we investigated whether CsA and Df, in conjunction with the antipsychotic medication CPZ, influence IL-1β expression in microglia. Transcription of the IL-1β gene and secretion of its gene product, which were enhanced by 25OHChol and 27OHChol, were attenuated in the presence of CsA and CPZ (Figure 6A,B). However, Df did not impact the oxysterols-induced expression of IL-1β. CsA inhibited the expression of IL-1β induced by 25OHChol and 27OHChol with an IC_50_ of 34.6 nM and 35.8 nM, respectively. CPZ inhibited the expression of IL-1β induced by 25OHChol and 27OHChol with an IC_50_ of 1.54 μM and 1.41 μM, respectively (Figure 6C,D). Furthermore, the immunoreactivity of MHC II on oxysterols-exposed microglia was reduced by treatment with CsA and CPZ, but not by Df (Supplementary Figure S5). These findings suggest that CsA and CPZ can modulate the oxysterol-induced inflammatory immune response and mitigate the overactivation of microglia.

## 3. Discussion

Previous studies suggested that 24sOHChol, 25OHChol, and 27OHChol have overlapping yet distinct roles in cholesterol metabolism. In this study, our primary objective was to explore the novel pathophysiological implications of oxysterols in the brain, with a specific focus on their impact on microglial activation. Excessive cholesterol within neurons undergoes oxidation to form 24sOHChol, catalyzed by cholesterol 24-hydroxylase (CYP46A1). This predominant oxysterol in the brain was shown to inhibit cholesterol synthesis and promote cholesterol efflux by activating liver X receptors (LXRs) in astrocytes [21]. In contrast, the majority of 27OHChol in the brain originates from the bloodstream, crossing the blood–brain barrier [22]. It facilitates cholesterol efflux from astrocytes by inducing the expression of the cholesterol transporter ABCA1 [23]. Notably, 25OHChol, primarily produced by macrophages, including microglia, curtails cholesterol synthesis in astrocytes [24]. Our study unveiled distinctive effects of side-chain oxysterols on microglial activation and IL-1β expression. Although 24sOHChol failed to activate microglia, we investigated its potential to suppress IL-1β expression. Our findings demonstrate that 24sOHChol did not inhibit IL-1β expression induced by 25OHChol and 27OHChol, suggesting that 24sOHChol is unlikely to modulate neuroinflammation triggered by these two oxysterols (data not presented). These discoveries contribute to an enhanced comprehension of the intricate roles played by oxysterols in brain function.

Upon exposure to pathogen-associated molecular patterns (PAMPs) and/or endogenous damage-associated molecular patterns (DAMPs), as well as the removal of immune-suppressive signals, microglia become activated [25]. However, chronic activation of microglia causes neuronal damage through the release of cytotoxic molecules such as pro-inflammatory cytokines, reactive oxygen intermediates, and proteinases [13]. Among the inflammatory cytokines released by activated microglia, IL-1β, in particular, was identified as a pivotal regulator of the local tissue response to brain injury and disease [26,27]. IL-1β not only mediates microglial activation and proliferation, but also induces neuronal cell death [28,29]. We demonstrated that 25OHChol and 27OHChol induce the production of IL-1β as well as the expression of MHC II, suggesting that extracellularly accumulated 25OHChol and 27OHChol molecules can function as DAMPs. Given that the overexpression of IL-1β can lead to chronic neuroinflammation and AD [30], the extracellular accumulation of side-chain oxysterols may contribute to neuroinflammatory responses and the progression of AD.

The increase in MHC II expression is a common feature of microglial activation. Under normal conditions, microglia exhibit low levels of MHC II protein or lack its expression altogether, but during periods of inflammation, its presence on the cell surface is induced [31]. Although the functional significance of surface MHC II in microglia and its role in the neuroinflammatory response remain unclear [15], we hypothesized that identifying protein markers, in addition to MHC II, specifically expressed in oxysterol-activated microglia, would be valuable for early AD diagnosis. Therefore, subsequent to confirming the surface expression of MHC II, we endeavored to identify other markers expressed in oxysterol-activated microglia. Our investigations revealed an increase in CD137 transcripts following treatment with 27OHChol, as demonstrated through RT-PCR (Supplementary Figure S6). However, we were unable to obtain conclusive data regarding the increase in CD137 protein expression, as assessed by immunofluorescence and Western blotting.

ApoE-deficient mice served as a widely utilized murine model for studying the progression of AD and atherosclerosis. These mice lack the ApoE protein, which plays a pivotal role in recognizing and clearing lipoproteins involved in cholesterol transport [32,33,34]. Consequently, they exhibit delayed clearance of lipoproteins and develop severe hypercholesterolemia. Even when maintained on a standard diet, these mice display elevated plasma cholesterol levels, which further escalate when subjected to a Western-type diet [35,36]. The absence of ApoE also disrupts cholesterol metabolism within the brain, as ApoE is primarily responsible for cholesterol transport in the central nervous system [37]. Elevated cholesterol levels within these mice lead to an increased production of oxysterols, including 25OHChol and 27OHChol [38]. Consequently, the consumption of a high-cholesterol diet results in significantly higher quantities of 25OHChol and 27OHChol formation compared to a standard chow diet. Moreover, 27OHChol has the ability to traverse the blood–brain barrier, accumulating within the brain [22]. This accumulation contributes to the presence of side-chain oxysterols in the brains of ApoE-deficient mice. The observed dysregulated cholesterol metabolism, particularly within the brain, in conjunction with markedly elevated blood cholesterol levels, highlights ApoE-deficient mice as an appropriate model for investigating the mechanisms that underlie microglial activation and neuroinflammation associated with hypercholesterolemia.

Dysregulated cholesterol metabolism emerged as a significant factor in the pathogenesis of neurodegenerative diseases [4,39]. AD, in particular, is associated with an elevated risk of high blood cholesterol levels. Studies unequivocally showed that increased total cholesterol levels during middle age independently elevate the risk of late-life AD, irrespective of the presence of the ApoE E4 allele or high systolic blood pressure [4,40]. Although the precise mechanisms remain incompletely understood, research utilizing mouse models fed a high-fat/cholesterol diet provided insights into the relationship between hypercholesterolemia and neuroinflammation. This neuroinflammatory response is characterized by the upregulation of pro-inflammatory cytokines such as TNF-α, IL-1β, and IL-6, as well as activation of glial cells, as evidenced by CD45 and GFAP immunostaining within the hippocampus [41,42]. Furthermore, this condition is associated with cognitive impairment, including a loss of working memory. In our study, we observed co-localization of IL-1β and MHC II immunoreactivities in microglia within the hippocampus of ApoE-deficient mice. These findings provide further support for the hypothesis that elevated cholesterol levels induce microglial activation and subsequent neuroinflammation, contributing to the pathogenesis of neurodegenerative diseases.

Signal transduction pathways mediate the conversion of extracellular stimuli into intracellular signals, thereby regulating gene expression. These pathways play a crucial role in the production of cytokines by immune cells, making them significant and appealing targets for therapeutic intervention in inflammatory diseases [43,44]. Consequently, one of our objectives was to identify the specific kinase pathway involved in the effects of side-chain oxysterols. Our findings reveal that the activation of microglia and the expression of IL-1β induced by 25OHChol and 27OHChol are mediated by the PI3K, ERK, and Src pathways. These results are consistent with a previous study that reported the involvement of various pathways in microglial cytokine production [45,46,47]. Since the PI3K, ERK, and Src pathways play critical roles in various cellular processes, such as cell growth, proliferation, survival, migration, secretion, and differentiation [48,49,50], dysregulated activity of these kinases affects several cellular responses. Therefore, we suggest that identifying the upstream signaling molecules responsible for immune oxysterol-induced activation of kinases is crucial for targeting neuroinflammation.

We conducted an experiment with the objective of identifying a pharmacological agent capable of mitigating the effects of immune oxysterols on microglia. In the course of this investigation, we serendipitously discovered a novel pharmacological action of CsA and CPZ. Our findings demonstrate that CsA and CPZ effectively inhibit microglial activation and the expression of IL-1β induced by 25OHChol and 27OHChol. Importantly, this inhibitory effect exhibits specificity, as the drugs did not impact cell viability at the concentrations required to suppress the effects of 25OHChol and 27OHChol (data not presented). CsA, an immunosuppressive agent derived from fungi, is widely utilized in the management of inflammatory disorders and organ transplantation. It exerts its immunosuppressive action by inhibiting T-cell activation [51,52]. Additionally, CsA demonstrates robust neuroprotective properties when it crosses the blood–brain barrier (BBB) and was proposed as a potential neuroprotective agent in cases of brain injury [53,54]. Conversely, CPZ is a pharmaceutical used to address diverse symptoms, including those associated with psychotic disorders such as schizophrenia, bipolar disorder, and asthma [55,56]. CPZ also possesses anti-serotonergic and antihistaminergic attributes [57]. In light of our results, we posit that CPZ and CsA may hold promise as therapeutic agents for neurodegenerative diseases characterized by neuroinflammation. Nonetheless, further in vivo studies are imperative to assess the therapeutic benefits of these compounds.

Based on the findings of this study, it can be concluded that elevated levels of side-chain oxysterols, such as 25OHChol and 27OHChol, resulting from dysregulated cholesterol metabolism, have the capacity to activate microglia, leading to the secretion of IL-1β through multiple kinase pathways. This activation of microglia and subsequent neuroinflammation may play a role in the development of neurodegenerative diseases, including AD. Targeting the mechanisms responsible for the effects of immune oxysterols through pharmacological interventions represents a potential strategy for mitigating neuroinflammatory disorders. A previous study documented that astrocytes exert an inhibitory effect on Aβ-induced neuronal damage [58]. The findings of this study highlight the importance of considering astrocytes as potential markers for treatment and mitigation strategies in AD. It is imperative to emphasize the necessity for future research endeavors aimed at integrating the insights gained from this study, which would elucidate the neuronal cytotoxicity resulting from microglial activation by oxysterols, in conjunction with the neuroprotective functions exhibited by astrocytes.

## 4. Materials and Methods

### 4.1. Cell Culture and Reagents

The HMC3 cell line was procured from the American Type Culture Collection (ATCC, Manassas, VA, USA). HMC3 microglia were cultured in Dulbecco’s Modified Eagle’s Medium (DMEM) supplemented with 10% fetal bovine serum (FBS) in the presence of penicillin and streptomycin. Cholesterol was obtained from Sigma-Aldrich (St. Louis, MO, USA), while 24sOHChol, 25OHChol, and 27OHChol were sourced from Santa Cruz Biotechnology (Santa Cruz, CA, USA). The Aβ_1-42_ was acquired from Abcam (Cambridge, UK). Primary antibodies against MHC II and the phosphorylated and unphosphorylated forms of ERK were obtained from Santa Cruz Biotechnology. Antibodies against the phosphorylated and unphosphorylated forms of Akt (Ser473) and Src (Tyr416) were purchased from Cell Signaling Technology (Danvers, MA, USA). CPZ, CsA, and Df were procured from ENZO Life Science, Inc. (Farmingdale, NY, USA).

### 4.2. Reverse Transcriptase and Real-Time Polymerase Chain Reaction (RT and Real-Time PCR)

Total RNA was reverse transcribed at 42 °C for 1 h using 100 U Moloney Murine Leukemia Virus (MMLV) reverse transcriptase in a 10 μL reaction volume containing 50 mM Tris-HCl (pH 8.3 at 25 °C, 55 mM KCl, 3 mM MgCl_2_, 10 mM DTT, 1 μg oligo dT 15 primers, 0.125 mM each dNTP, and 40 U RNase inhibitor). The cDNA was amplified for 27 cycles at 95 °C for 30 s, 55 °C for 30 s, and 72 °C for 30 s. PCR products were detected on an EtBr-containing agarose gel. Quantitative real-time PCR (qPCR) was performed as previously described [19]. Briefly, the reactions of qPCR were performed in triplicate using a LightCycler^®^ 96 Real-Time PCR System (Roche, Germany); each 20 μL reaction consisted of 10 μL of SYBR Green Master Mix, 2 μL of forward and reverse primers (10 pM each) of genes to be analyzed, and a cDNA template. Thermal cycling conditions were as follows: 95 °C for 10 min, 45 cycles at 95 °C for 10 s, 50 °C for 10 s, and an elongation period of 10 s at 72 °C. The primer sequences for qPCR were IL-1β: 5-’-TGAGCTCGCCAGTGAAATGA-3′ (forward) and 5′-AGATTCGTAGCTGGATGCCG-3′ (reverse); GAPDH: 5′-ATGGGGAAGGTGAAGGTCG-3′ (forward) and 5′-GGGGTCATTGATGGCAACAATA-3′ (reverse). The relative expression of the gene of interest was calculated as a ratio to GAPDH using LightCycler 96 software (Version 1.1.0.1320).

### 4.3. Western Blot Analysis

HMC3 microglia were lysed using Pro-Prep^TM^ lysis buffer (Intron Biotechnology, Sungnam, Republic of Korea). Equal amounts of protein from each sample were separated by SDS-PAGE and transferred onto a nitrocellulose membrane. After blocking the non-specific binding of the primary antibody with 5% skim milk in 0.1% Tween 20/TBS, the membrane was probed with indicated primary antibodies at 4 °C overnight. Subsequently, the membrane was washed thrice with 0.1% Tween 20/TBS for 15 min each and incubated with HRP-conjugated secondary antibodies for 1 h at room temperature. After washing three times with washing buffer for 15 min each, bands were detected with a PIERCE^®^ enhanced chemiluminescence Western blotting detection system (Thermo Scientific, Rockford, IL, USA). Chemiluminescent images were captured using an Amersham Imager 600 (GE Healthcare Life Sciences, Pittsburgh, PA, USA).

### 4.4. ELISA

The amounts of IL-1β secreted from cells into the culture medium were quantitatively measured using a commercially available ELISA kit (BD OptEIA^TM^ Human IL-1β ELISA Kit II) according to the manufacturer’s instructions (BD Biosciences, San Diego, CA, USA).

### 4.5. Immunofluorescence of MHC II

HMC3 cells were cultured on gelatin-coated coverslips (0.2% gelatin in PBS) and stimulated with cholesterol or oxysterol for 48 h. The cells were fixed with 1% paraformaldehyde for 20 min and incubated with a blocking solution of 5% skim milk in PBS for 1 h. Subsequently, the cells were incubated at room temperature for 2 h with a primary antibody against MHC II, which was diluted 1:100 in the blocking solution. The cells were washed twice with PBS for 5 min each and incubated at room temperature in the dark for 1 h with fluorescence-conjugated secondary antibodies, which were diluted 1:200 in PBS. Following another wash with PBS, the cells stained with fluorescence were mounted and visualized using a confocal microscope (FV1000; Olympus Corp., Tokyo, Japan).

### 4.6. Immunofluorescence Staining of Murine Brain Sections

Animal experiment protocols were approved by the Institutional Animal Care and Use Committee of the School of Medicine, Pusan National University, and the experiment was performed in accordance with the Korean Food and Drug Administration (KFDA) guidelines (Approval number: PNU-2022-0257). *ApoE*-deficient mice (8-week-old, males) were subjected to an 8-week feeding regimen with a HFD containing cholesterol (7.5% cocoa butter and 1.25% cholesterol), as previously described [17]. Following the 8-week feeding period, the mice were euthanized by inhalation of carbon dioxide. Whole brains were immediately isolated from the murine skulls, fixed in 4% paraformaldehyde for 48 h at 4 °C, and transferred to 30% sucrose solution. The brains were then cut into 10 μm sections using a cryostat microtome (Leica CM 1850; Leica Microsystems, Seoul, Republic of Korea). The brain sections were biotinylated with anti-IL-1β, MHC II (Invitrogen, Waltham, MA, USA, #14-5321-82), and Iba-1 (Wako, #019-19741) antibodies (1:100) by incubating overnight at 4 °C and washed three times in PBS for 10 min each. Following the washing step, the sections were incubated for 1–2 h at room temperature with anti-rat secondary antibodies conjugated to Alexa Fluor 488 nm and anti-rabbit secondary antibodies conjugated to Alexa Fluor 568 nm (Invitrogen-Molecular Probes, Eugene, OR, USA). The sections were then washed twice with PBS for 10 min each, followed by a wash in distilled water for 10 min, and mounted with VectaShield^TM^ mounting media with DAPI. After incubating overnight at 4 °C without light, immunofluorescence images were acquired using a K1-Fluo laser scanning confocal microscope (Nanoscope Systems, Daejeon, Republic of Korea).

### 4.7. Statistical Analysis

Statistical analyses were performed using one-way ANOVA, followed by Dunnett’s multiple comparison test, using GraphPad PRISM (version 5.0; GraphPad Software Inc., San Diego, CA, USA).

## Data Availability

All data generated or analyzed during this study are included in this published article and its supplementary information files.

## Supplementary Materials

The supporting information can be downloaded at: https://www.mdpi.com/article/10.3390/ijms242015288/s1.
