# Supplementary material for: Side-Chain Immune Oxysterols Induce Neuroinflammation by Activating Microglia
Source: Int J Mol Sci. 2023 Oct 18;24(20):15288. doi: 10.3390/ijms242015288 (PMC10607006; doi:10.3390/ijms242015288)
Supplement: Supplementary file 1 [file ijms-24-15288-s001.zip › ijms-2644172-supplementary.pdf]

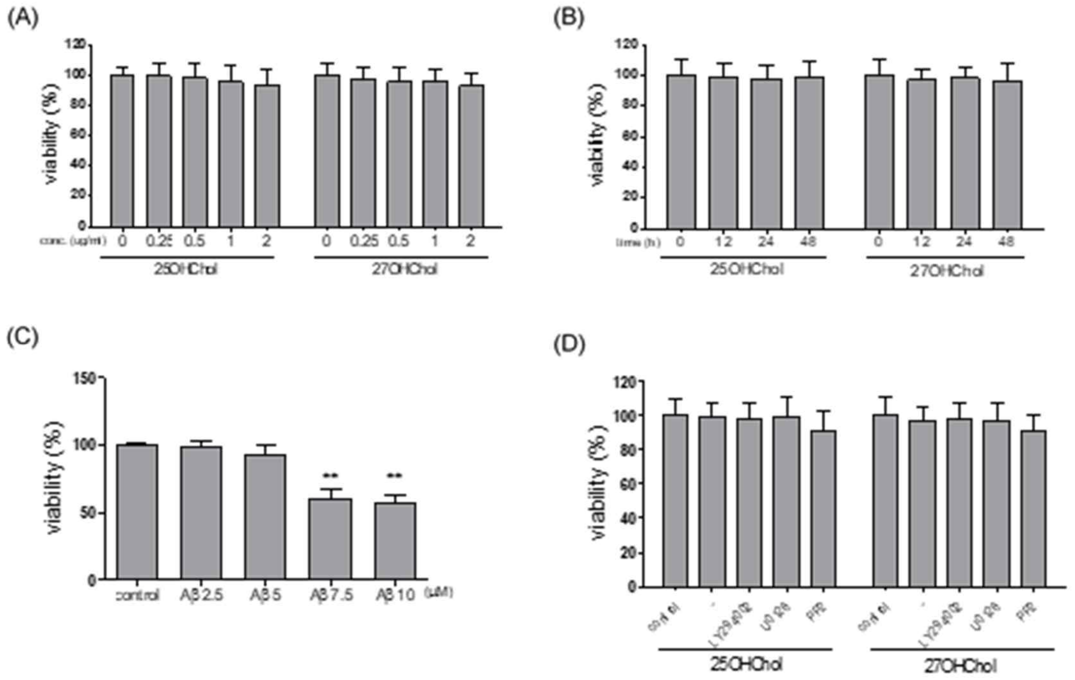

Supplementary Figure S1. Viability of HMC3 cells.

HMC3 cells (5 × 10^3 cells/well) were treated for 48 h with the indicated concentrations of 25OHChol and 27OHChol (A) or for the indicated time periods with 25OHChol or 27OHChol (1 μg/ml each) (B). HMC3 cells (5 × 10^3 cells/well) were treated with various concentrations of $A\beta_{1-42}$ for 48 h (C), and the cells were treated for 48 h with 25OHChol or 27OHChol (1 μg/ml each) in the absence or presence of the indicated inhibitors (D). After the treatments, cell viability was determined using cell counting kit-8 (CCK-8; Dojindo Molecular Technologies, Inc., MD20850, USA). The results are representative of three independent experiments. ** $P < 0.01$ vs. control.

HMC3, human microglial clone 3 cell line.

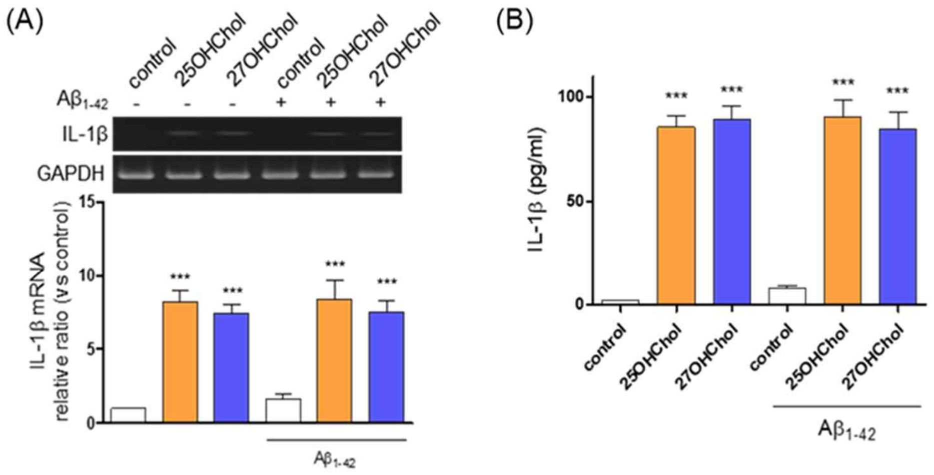

Supplementary Figure S2. Effects of A$\beta_{1\text{-}42}$ and side-chain immune oxysterols on the IL-1β expression in microglial cells.

Microglia were treated for 48 h with A$\beta_{1\text{-}42}$ (5 μM) in the absence or presence of 25OHChol or 27OHChol (1 μg/ml each). After isolation of total RNA from the cells, IL-1β transcripts were detected by RT-PCR and its levels were assessed by qPCR (A). The amount of IL-1β released into culture media were quantitatively detected by ELISA (B). *** $P < 0.001$ vs. control. Data are expressed as the mean±SD (n=3 replicates for each group).

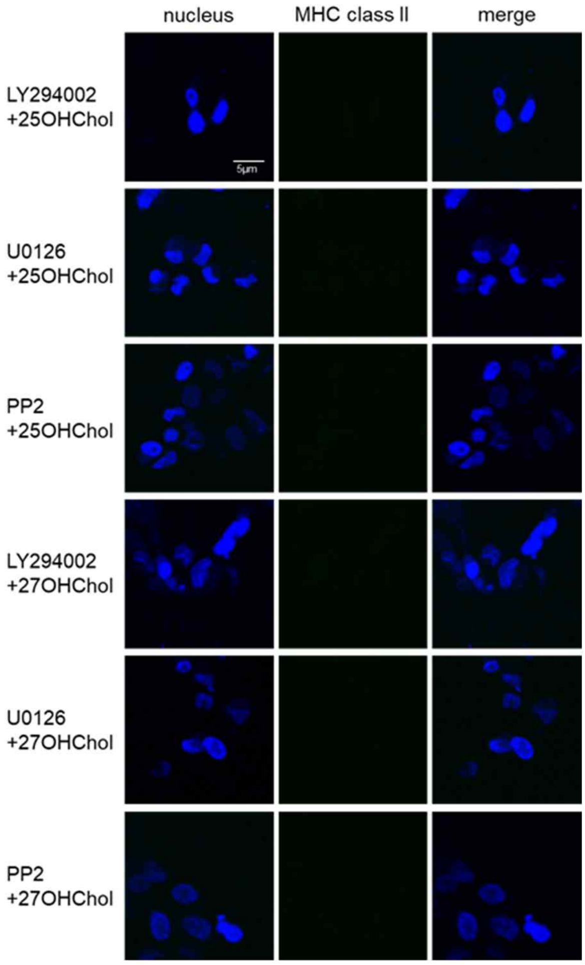

Supplementary Figure S3. Images of MHC II following treatment with kinase inhibiters.

HMC3 cells were seeded on coverslip and treated for 48 h with 25OHChol and 27OHChol (1 μg/ml each) in the presence of indicated inhibitors (10 μM each). After immunostaining MHC class II with fluorescence-conjugated antibodies (green), the fluorescence was visualized by confocal microscopy (200x). The nuclei were stained with DAPI (blue). Results are representative of three independent experiments.

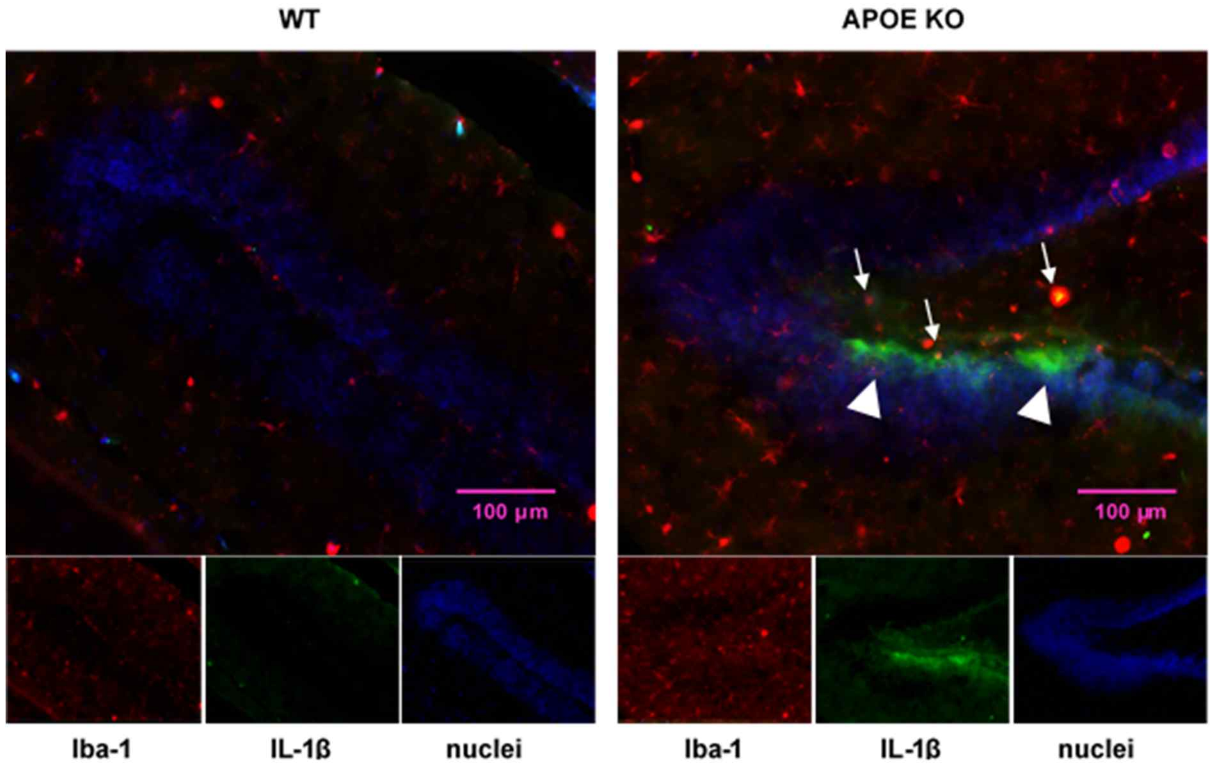

Supplementary Figure S4. Images of IL-1β in the hippocampus of ApoE-deficient mice.

The brain sections were prepared from wild-type and ApoE-deficient mice fed a high-cholesterol diet and immunostained with fluorescent dye-conjugated antibodies. The sections were labelled with antibodies against Iba-1 (red) and anti-IL-1β (green). The nuclei were stained with DAPI. Co-localized images are indicated by white arrows, and white arrowheads indicate released IL-1β. The results are representative of three independent experiments. Scale bars represent 100 µm.

IL-1β, interleukin-1β.

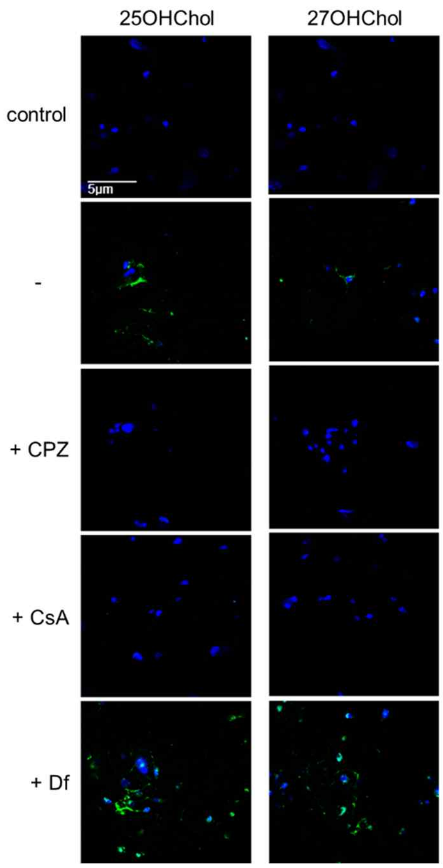

Supplementary Figure S5. Effects of CPZ and CsA on expression of MHC II.

HMC3 cells were seeded on coverslips and treated for 48 h with 25OHChol or 27OHChol (1 μg/ml each) in the absence or presence of CPZ (2μM), CsA (50 nM) or Df (25 μg/ml). The cells were immunostained with fluorescence-conjugated antibody for MHC class II (green), and the nuclei were stained with DAPI (blue). The fluorescence was visualized by confocal microscopy (200x). Results are representative of three independent experiments.

CPZ, chlorpromazine: CsA, cyclosporin A; Df, dichlofenac.

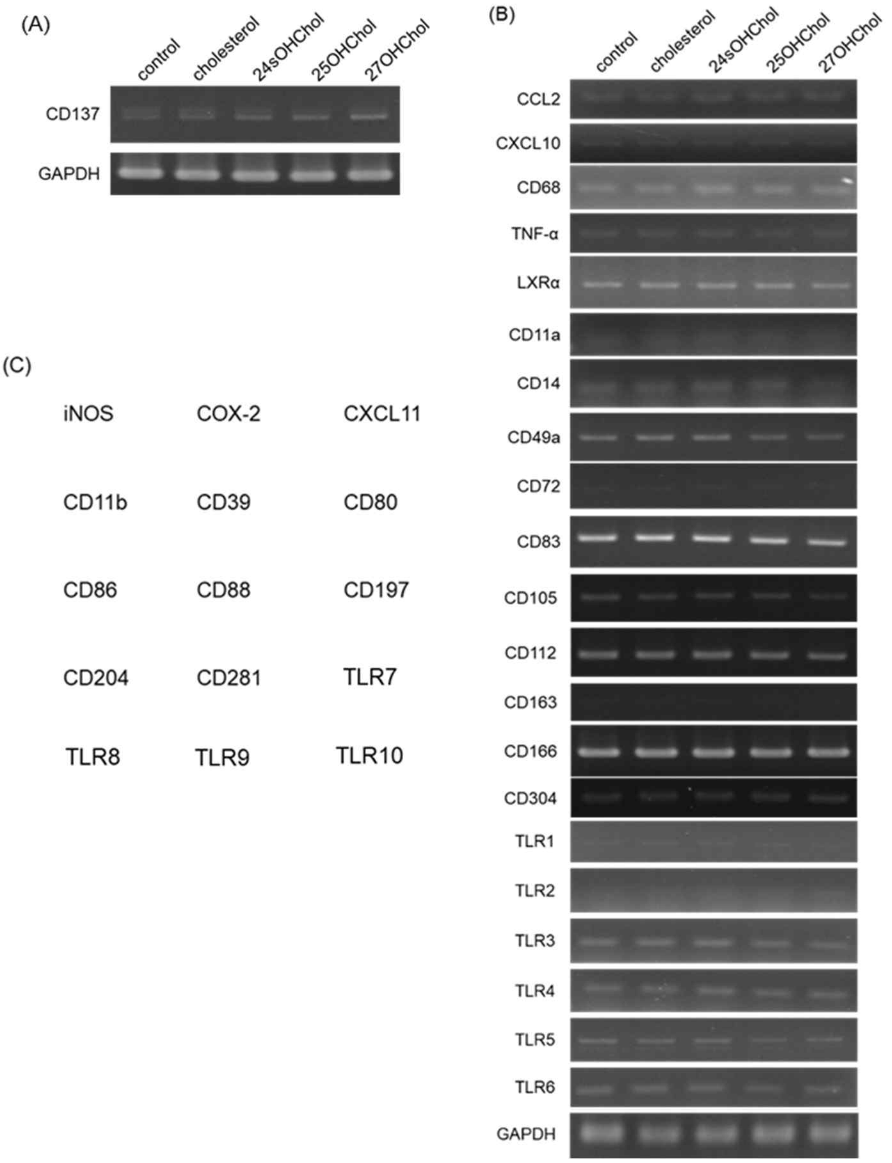

Supplementary Figure S6. Effects of side-chain oxysterols on M1/M2 markers and immune-modulatory factors in HMC3 cells.

HMC3 microglial cells were treated for 48 h with cholesterol and the indicated side-chain oxysterols (1 μg/ml each). Using the total RNA isolated from the cells, and expression of M1/M2 markers and CD molecules were detected by RT-PCR. The genes analyzed were as follows: (A) CD137, (B) the genes whose transcript levels were not changed, and (C) the genes whose transcripts were not detected. The results are representative of three independent experiments.

HMC3, human microglial clone 3 cell line.
